# Exploring Next-Generation Engineering Bioplastics: Poly(alkylene furanoate)/Poly(alkylene terephthalate) (PAF/PAT) Blends

**DOI:** 10.3390/polym11030556

**Published:** 2019-03-23

**Authors:** Niki Poulopoulou, Nejib Kasmi, Maria Siampani, Zoi N. Terzopoulou, Dimitrios N. Bikiaris, Dimitris S. Achilias, Dimitrios G. Papageorgiou, George Z. Papageorgiou

**Affiliations:** 1 Chemistry Department, University of Ioannina, P.O. Box 1186, 45110 Ioannina, Greece; nikki_p@windowslive.com (N.P.); mariasiampani16@gmail.com (M.S.); 2 Laboratory of Polymer Chemistry and Technology, Department of Chemistry, Aristotle University of Thessaloniki, GR-541 24 Thessaloniki, Macedonia, Greece; nejibkasmi@gmail.com (N.K.); terzoe@gmail.com (Z.N.T.); dbic@chem.auth.gr (D.N.B.); axilias@chem.auth.gr (D.S.A.); 3 School of Materials and National Graphene Institute, University of Manchester, Oxford Road, Manchester M13 9PL, UK

**Keywords:** dynamic homogeneity, poly(alkylene 2,5-furandicarboxylate)s, poly(alkylene terephthalate)s, polymer blends, reactive blending

## Abstract

Polymers from renewable resources and especially strong engineering partially aromatic biobased polyesters are of special importance for the evolution of bioeconomy. The fabrication of polymer blends is a creative method for the production of tailor-made materials for advanced applications that are able to combine functionalities from both components. In this study, poly(alkylene furanoate)/poly(alkylene terephthalate) blends with different compositions were prepared by solution blending in a mixture of trifluoroacetic acid and chloroform. Three different types of blends were initially prepared, namely, poly(ethylene furanoate)/poly(ethylene terephthalate) (PEF/PET), poly(propylene furanoate)/poly(propylene terephthalate) (PPF/PPT), and poly(1,4-cyclohenedimethylene furanoate)/poly(1,4-cycloxehane terephthalate) (PCHDMF/PCHDMT). These blends’ miscibility characteristics were evaluated by examining the glass transition temperature of each blend. Moreover, reactive blending was utilized for the enhancement of miscibility and dynamic homogeneity and the formation of copolymers through transesterification reactions at high temperatures. PEF–PET and PPF–PPT blends formed a copolymer at relatively low reactive blending times. Finally, poly(ethylene terephthalate-*co*-ethylene furanoate) (PETF) random copolymers were successfully introduced as compatibilizers for the PEF/PET immiscible blends, which resulted in enhanced miscibility.

## 1. Introduction

Polymeric materials offer a number of advantages that enable their extensive use in numerous everyday applications. Over the past few years, public awareness for sustainable packaging has increased, and as a result, the significance of bioplastics as an alternative to fossil-based plastics has been considered. The worldwide production of bioplastics, which was <1% before 2015, has been on the rise over the past few years [1]. The term “bioplastics” refers to either a polymer that is produced from renewable resources, or to one which is biodegradable or compostable at its end-of-life [2]. Currently, one of the most popular classes of polymers is the thermoplastic polyester, which are used in the production of fibers, films, and others for applications in construction, transportation, packaging, and engineering. The global demand for polyesters is increasing at an average of 4% per year [3]. Poly(alkylene terephthalate)s (PATs) dominated the market for decades, and their most prominent member, poly(ethylene terephthalate) (PET), shows beneficial thermal, mechanical, and gas barrier properties at a reasonable cost. The annual production of PET is estimated to be about 56 million tonnes [4]. 

At present, there is a trend for strong, non-biodegradable biopolymers that can accommodate a number of applications, such as bio-poly(ethylene terephthalate) (bio-PET). The global biobased PET market is expected to grow by up to 68% from 2015 to 2019. The key issue in bio-PET production is to establish a sustainable production of monomers from renewable resources derived from biomass [5]. 

In 2009 the Coca-Cola Company successfully replaced ethylene glycol (EG) that originated from petroleum-derived sources with EG from biobased sources, and thus introduced the bio-PET bottle or “PlantBottle”. Moreover, several companies are seeking to manufacture biobased terephthalic acid (TA) [6]. A different approach is to replace petroleum-based TA with biobased 2,5-furandicarboxylic acid (FDCA), which can be obtained by the oxidation of hydroxymethyl-furfural (HMF), which in turn can be derived by the dehydration of carbohydrates such as fructose [7,8,9]. This means the replacement of PET by its biobased counterpart, poly(ethylene 2,5-furandicarboxylate) (PEF) [10,11]. 

Polyesters based on FDCA and poly(alkylene furanoate)s (PAFs) such as PEF, besides their green nature, show superior gas barrier, mechanical, and thermal properties compared to their terephthalate homologues. For example, amorphous PEF exhibits an 11-fold reduction in permeability in oxygen, 19-fold in carbon dioxide, a 5-fold reduction in water diffusion, and a 2.8-fold water permeability reduction (at 35 °C) compared to amorphous PET [12,13,14]. 

Furanoate and terephthalate polyesters show similarities in their structures as they are based on FDCA and TA, respectively. On the other hand, the main differences between the two lie in their ring size, geometry, and polarity [15,16]. In TA the interatomic distance among carboxylic acid groups is 5.731 Å, compared to the much shorter one of 4.830 Å in FDCA [17]. In the case of TA, the carbon atoms of the carboxyl units are linear, in contrast to the nonlinear structure of FDCA with an angle of 129.4°. The crystallization is much slower in the case of furanoates compared to terephthalates because of the nonlinearity and the permanent dipole in FDCA [18,19,20,21]. Finally, a larger density value has been reported (ρ = 1.4299 g/cm^3^) for amorphous PEF compared to that of 1.3346 g/cm^3^ for PET [22]. Among PAFs, PEF, poly(propylene furanoate) (PPF) or poly(trimethylene furanoate) (PTF), poly(butylene furanoate) (PBF), and poly(1,4-cyclohexanedimethylene furanoate) (PCHDMF) have attracted the interest of both academia and the bioplastic industry [23,24,25,26,27,28,29,30,31,32]. 

Blending is one of the three main ways to obtain polymeric materials with tailor-made properties, the other two being the synthesis of novel polymers and the modification of the structure of existing materials [33]. In contrast with furanoate-based blends, the respective blends of PET, poly(propylene terephthalate) (PPT), and poly(butylene terephthalate) (PBT) have been studied extensively [34,35,36]. Reactive blending is a method for increasing homogeneity and thermodynamic miscibility in polymer blends, and it involves the heating and mixing of the two polymers at temperatures well above their melting temperatures. Transesterification reactions take place under such conditions, giving rise to the formation of block and, finally, random copolymers depending on the time and temperature of melt mixing [37]. Currently, two studies have been published in the literature on PBF–PLA blends [38,39], and some preliminary results on all furan-based blends have been published by our group [37].

In this work, three different series of polymer blends, namely, PEF/PET, PPF/PPT, and PCHDMF/poly(1,4-cycloxehane terephthalate) (PCHDMT) blends were prepared by solution blending and studied for the first time. Solution blending was initially selected for the preparation the blends in order to avoid any possible transesterification reactions. A number of low-mass samples were first prepared to test all three series of blends over the whole composition range by using small lab-synthesized quantities of the novel furanoate polyesters. The motivation for this work was the fact that even though furanoates show improved properties compared to terephthalates, the existing industrial infrastructures are designed for terephthalate production and cannot be used for furanoate production unless they are modified. 

However, with blending, materials with balanced properties and cost can be produced in order to meet the demands of different applications. Furthermore, furanoates crystallize very slowly, and therefore blending with terephthalates can be an efficient approach for improving the crystallization rates of furanoates. Th is one of the most important issues that needs to be addressed before furanoates can be produced in large volumes for industrial applications.

## 2. Materials and Methods

### 2.1. Synthesis of Polyesters

A series of high-molecular-weight poly(alkylene furanoate) and poly(alkylene terephthalate) samples were synthesized by applying melt polycondensation as described in our previous studies [23,24].

**Figure 1:**
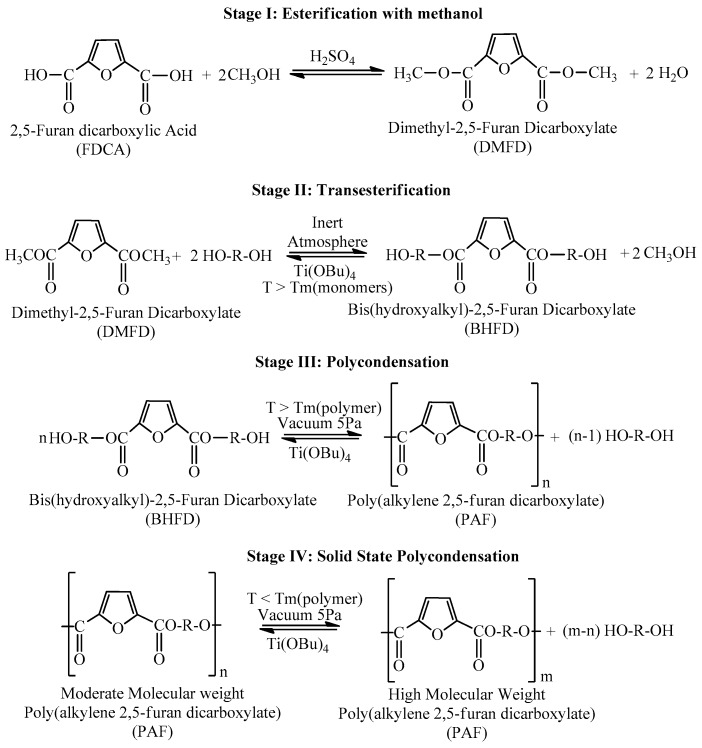
Reaction path for the synthesis of poly(alkylene 2,5-furan dicarboxylate)s studied in this work.

Dimethyl terephthalate (DMT) was obtained from Du Pont De Nemours Co (Hamm, Germany) and 2,5-furan dicarboxylic acid (purum 97%) was purchased from Sigma-Aldrich Chemical Co (Chemie GmbH, Hamburg, Germany). Tetrabutyl titanate (TBT) catalyst of analytical grade; 1,2-ethanediol, 1,3-propanediol, both of analytical grade; and 1,4-cyclohexanedimethanol (CHDM, 70/30 trans/cis, purum 99%), used as diols for polyester synthesis, were purchased from Sigma–Aldrich Chemical Co (Chemie GmbH). All other materials and solvents used were of analytical grade. Solid-state polycondensation (SSP) was subsequently applied to produce polymers of high molecular weight. The reaction path for the synthesis of the polyesters is shown in Figure 1.

### 2.2. Preparation of Polymer Blends

Polymer blends of the thermoplastic polyesters were prepared by dissolving the corresponding polymers in a mixture of trifluoroacetic acid and chloroform (4/1, *v*/*v*). The solutions were poured into an excess of methanol and the blends were obtained as a precipitate. Several blends with varying compositions were prepared. Solution mixing was selected for the preparation of blends in order to avoid any possible transesterification reactions occurring at elevated temperatures during melt mixing. The mixing procedure took place at ~45 °C for almost 2 hours.

### 2.3. Characterization Methods

#### 2.3.1. Intrinsic Viscosity Measurements

Intrinsic viscosity [η] measurements were performed using an Ubbelohde viscometer at 30 °C in a mixture of phenol/1,1,2,2-tetrachloroethane (60/40, *w*/*w*). The IV values were 0.61 g/dL for PEF, 0.67 g/dL for PPF, 0.46 g/dL for PCHDMF, 0.62 g/dL for PET, 0.71 g/dL PPT, and 0.49 g/dL for PCHDMT. 

#### 2.3.2. Differential Scanning Calorimetry

The thermal behavior of the blends was studied using a Perkin Elmer Diamond DSC upgraded to DSC 8500, combined with an Intracooler IIP cooling system. Samples of about 5 mg were used. The blends were first heated at 20 °C/min and increased up to 30 °C above the highest melting temperature and then quenched to −30 °C before reheating at a rate of 20 °C/min to observe the glass transition, cold-crystallization, and melting of the amorphous samples. For polyesters, reactive blending is an industrial process that involves melt mixing in an extruder/internal mixer at temperatures higher than the melting temperatures of all constituents. To simulate reactive blending, the blends were initially prepared from solution, as described above, and were subsequently melt-mixed inside the DSC pans. More specifically, for reactive blending experiments, the blends were scanned at a rate of 20 °C/min up to a predetermined temperature that was well above the melting points of both components, where they were held for a specific time in each test before quenching to −30 °C. The quenched samples were subsequently heated at 20 °C/min, starting from a temperature of at least 30 °C below the lower *T*_g_ of the polymers. For the evaluation of the glass transition, tangents were drawn carefully on the heat flow curve at temperatures above and below the glass transition and the *T*_g_ was obtained as the point of intersection of the bisector of the angle between the tangents with the heat flow curve. The intersection of these tangents with the part corresponding to the transition were used as *T*_g,onset_ and *T*_g,end_.

#### 2.3.3. X-Ray Diffraction

X-ray diffraction (XRD) measurements of the samples after grinding were performed using a SIEMENS Diffract 500 system (Munich, Germany) employing CuKα radiation (λ = 1.5418 Å). The samples that were tested were obtained after precipitation from the solution, hence they displayed a certain degree of crystallinity.

## 3. Results and Discussion

### 3.1. PEF–PET Blends

**Figure 2:**
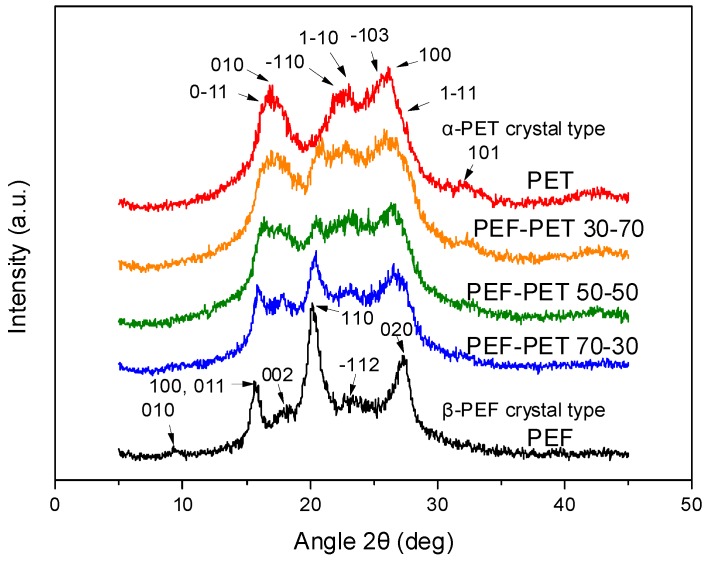
XRD patterns of poly(ethylene furanoate) (PEF), poly(ethylene terephthalate) (PET) and their blends.

The XRD patterns of the PEF–PET blends can be seen in Figure 2. The pattern of PEF corresponded to the β-crystal type of PEF [40]. This is reasonable, as the solution/precipitation method was applied for the preparation of these particular samples. It has been shown that PEF crystallizes in the β-crystal form when crystallized from solution [40]. For PET, α-type crystals were found to form [41]. The patterns of the blends show that both polymers crystallized, and mixtures of their crystals were present in the blends. 

**Figure 3:**
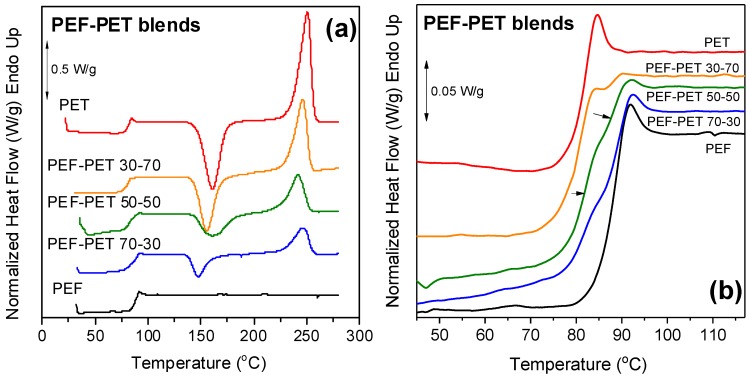

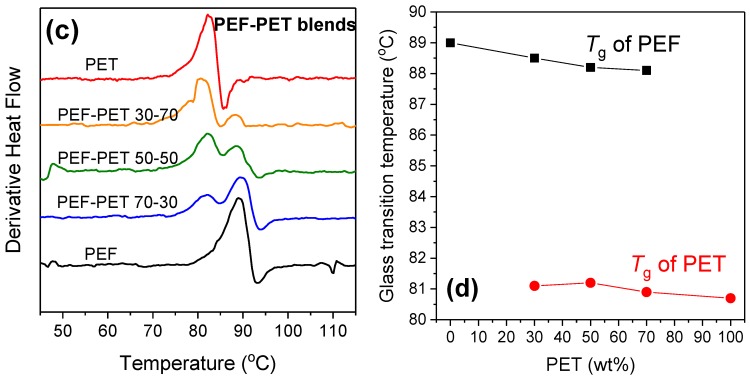
(**a**) DSC thermograms of PEF, PET, and their blends, (**b**) zoomed-in area of the glass temperature, (**c**) derivative of heat flow of the samples under study, and (**d**) the variation of *T_g_* with PET content.

The DSC traces of the melt-quenched PEF–PET blends are shown in Figure 3a, where double glass transitions can be observed. The glass transition temperatures of the two polymers were quite close: *T*_g_ = 88 °C for PEF and 81 °C for PET (Δ*T*_g_ = *T*_gPPF_ − *T*_gPPT_ = 88 – 81 = 7 °C). As a result, the corresponding signals overlapped within the glass temperature range. A magnified region of the glass temperatures of the blends can be seen in Figure 3b. The double transitions were rather clear, indicating a two-phase system. At this point it must be noted that a sensitive power compensation DSC was used in this study in order to enable the observation of contributions from both polyesters in the blends.

PEF could not crystallize during the heating scan at 20 °C/min. In contrast to PET, PEF is a slowly crystallizing polyester, especially when it possesses high molecular weight. The blends showed a tendency to crystallize less with increasing PEF content as the only component contributing to the phenomenon was PET. 

Study of the derivative of heat flow can lead to important conclusions. Figure 3c presents the derivative heat flow curves against temperature. Double peaks corresponding to the two glass transitions appeared in the thermograms of the blends, verifying the earlier hypothesis that was made based on the DSC thermograms. Finally, the fact that the *T*_g_s of PEF and PET remained practically unchanged among the different compositions of the blends demonstrates that the blends can be considered immiscible (Figure 3d).

### 3.2. PPF–PPT Blends

**Figure 4:**
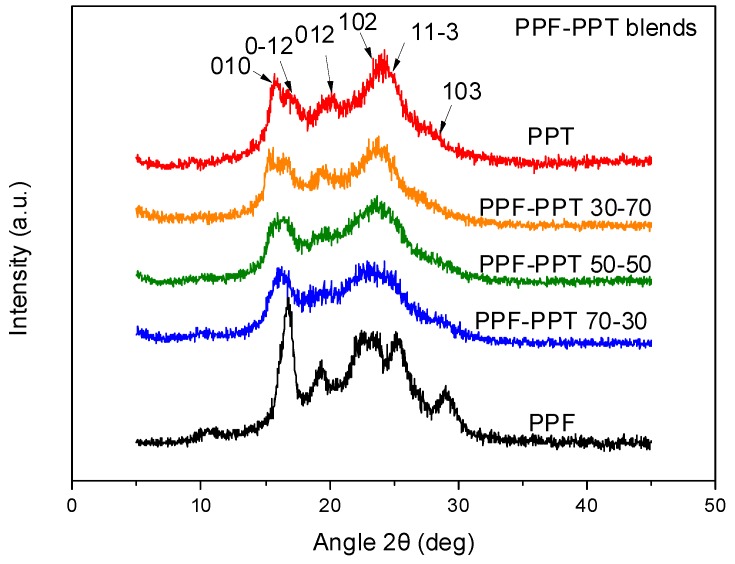
XRD patterns for neat poly(propylene terephthalate) (PPT), neat poly(propylene furanoate) (PPF), and their blends.

The crystal unit cell parameters for PTT are *a* = 4.64 Å, *b* = 6.27 Å, *c* = 18.64 Å, *a* = 98°, *b* = 90°, and *g* = 111° [42]. Unfortunately, there are no data available for the crystalline structure of PPF yet. As can be seen in Figure 4, crystal reflections for both components appeared in the XRD patterns for PPF–PPT blends. A second characteristic of the XRD patterns was a reduction in the intensity of the peaks in the blends, reflecting a decrease in the degree of crystallinity.

**Figure 5:**
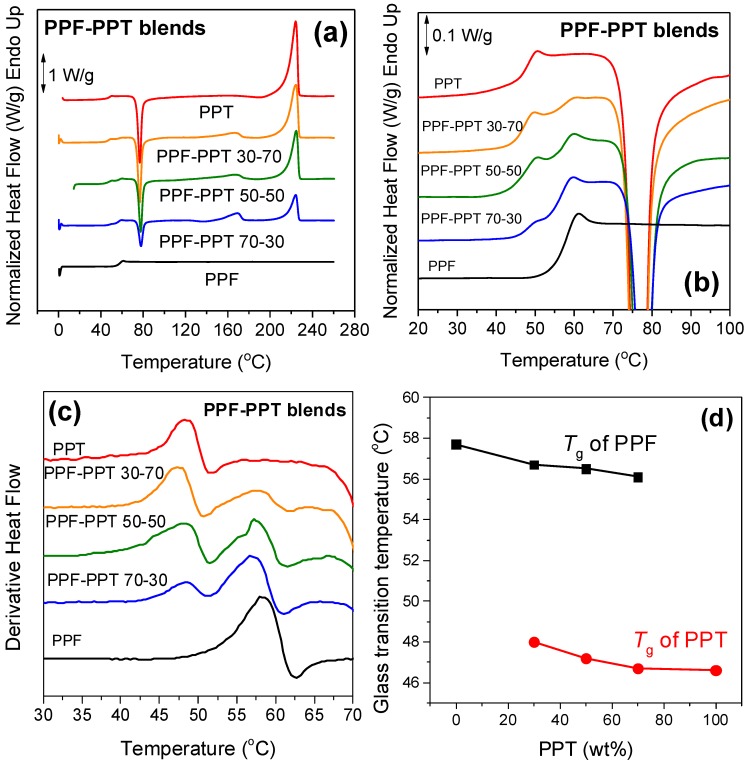
(**a**) DSC thermograms of PPF, PPT, and their blends, (**b**) zoomed-in area of the glass temperature, (**c**) derivative of heat flow of the samples under study, and (**d**) the variation of T_g_ with PPT content.

Two glass transitions can be observed in the DSC traces of the melt-quenched PPF–PPT blends shown in Figure 5a. The large difference in the crystallization rates of the two polymers can also be seen. PPT crystallized quickly, and this resulted in a sharp cold-crystallization peak at about 80 °C. On the other hand, PPF could not crystallize upon heating from the glassy state at a rate of 20 °C/min. However, a melting peak for PPF was evidenced in the DSC traces of the blends at 168 °C, even for low PPF content (30 wt %). This is important as it reveals that the addition of a small amount of PPT improved the crystallization characteristics of PPF, where it is well-known to crystallize at slow rates, and this is considered one of the major drawbacks for its mass production and subsequent use in applications.

In Figure 5b, where details of the glass transition region can be seen, the double glass transitions are clearer. Two peaks corresponding to the different glass transitions were observed in the curves of the derivative heat flow in Figure 5c. As the two *T*_g_s differed by *T*_gPPF_ – *T*_gPPT_ = 58 – 46 = 11 °C, the double peaks were well resolved compared to the PEF–PET blends. In any case, the use of the derivate of heat flow here is very important to better understand the behavior of the blends. Finally, the small variation in the *T*_g_ values with PPT content can be seen in Figure 5d. The above observations can once again be indicative of the formation of immiscible furanoate/terephthalate blends.

### 3.3. PCHDMF–PCHDMT Blends

**Figure 6:**
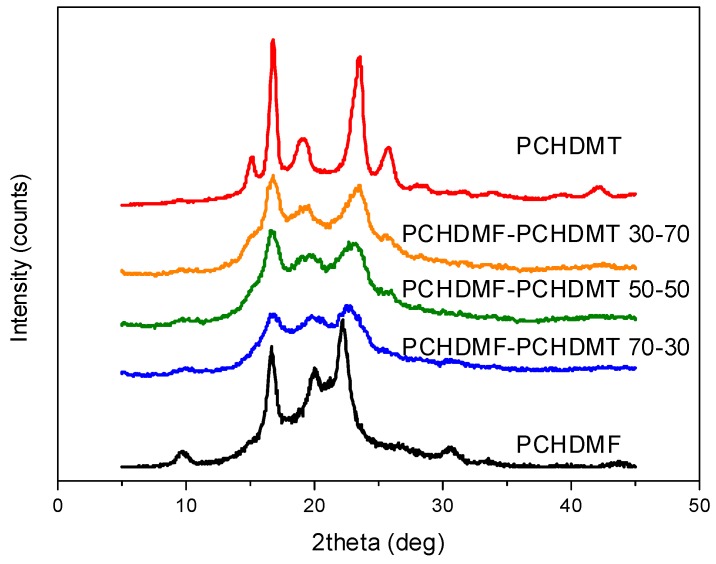
XRD patterns for neat poly(1,4-cyclohenedimethylene furanoate) (PCHDMF), poly(1,4-cycloxehane terephthalate) (PCHDMT), and their blends.

The last set of blends that were prepared and examined were the PCHDMF–PCHDMT blends. Both parent polymers are fast crystallizing. The blending procedure resulted in a lower degree of crystallinity in the blends, as seen in Figure 6.

**Figure 7:**
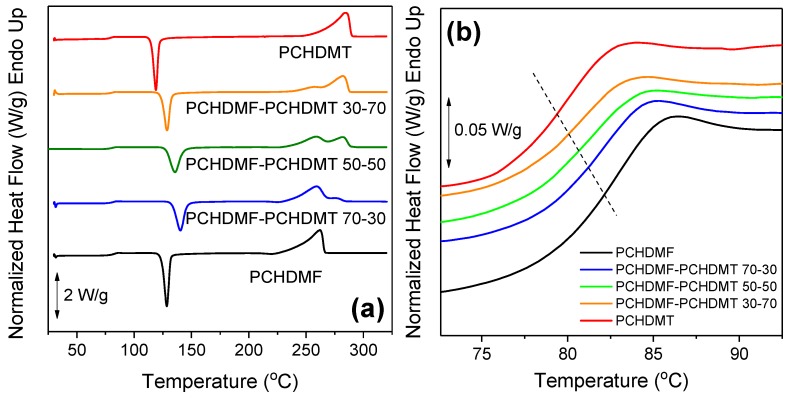

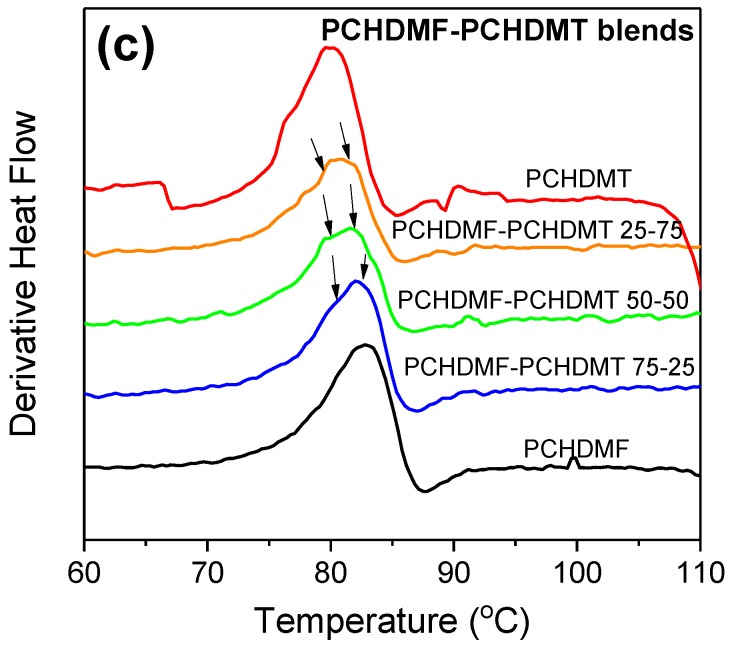
(**a**) DSC thermograms of neat PCHDMF, neat PCHDMT, and their respective blends, (**b**) zoomed-in area of the glass temperature, and (**c**) derivative of heat flow for all samples under study.

Concerning the glass transition behavior of the blends, it should be noted that for PCHDMF–PCHDMT the difference in the glass transition temperatures was very small: Δ*T*_g_ = *T*_gPCHDMF_ – *T*_gPCHDMT_ = 83 – 80 = 3 °C. On the other hand, there was a significant difference in the melting temperatures of the polymers (Δ*T*_m_ = *T*_mPCHDMT_ – *T*_mPCHDMT_ = 293 – 265 = 28 °C). Similar to the neat polymers, the blends showed cold-crystallization upon heating from the glassy state (Figure 7a). It seems that the blending procedure depressed the crystallization of both components, as the cold-crystallization temperature increased in all the blends compared to the neat polyesters. However, both components crystallized successfully, as revealed by the double melting peaks that appeared in the DSC traces. In the curves of Figure 7b, the decrease in *T*_g_ with increasing PCHDMT content in the blends was obvious, along with a broadening and a change in the slope of the signal in the glass transition region. This broadening was also detectable in the derivative heat flow signal of the blends. Double peaks also appeared in the derivative heat flow (Figure 7c) for the blends, which were quite pronounced—especially in the case of the PCHDMF–PCHDMT 50–50 sample. 

### 3.4. Reactive Blending

**Figure 8:**
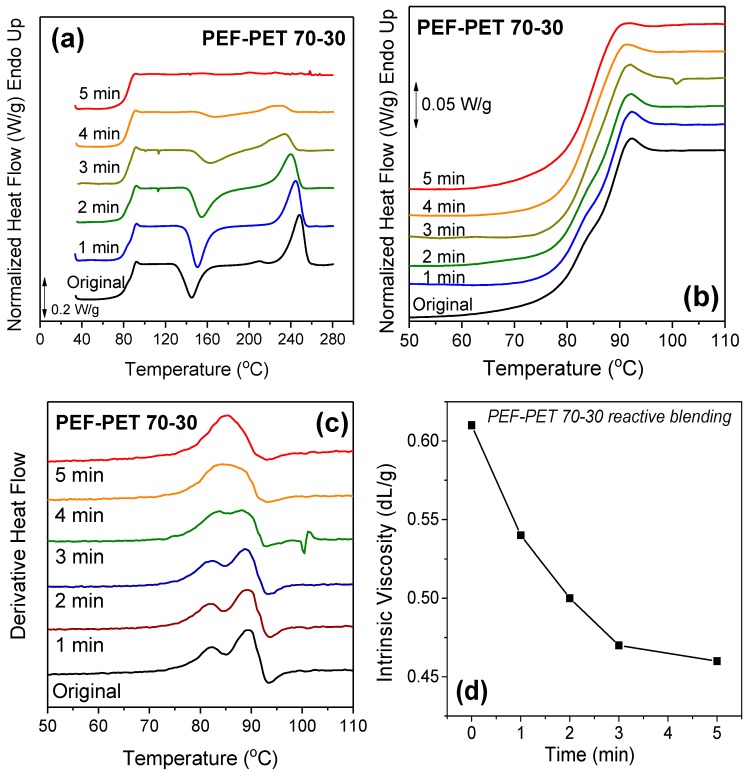
(**a**) Effect of time of reactive blending at 280 ℃ on the DSC thermograms of the PEF–PET 70–30 blends, (**b**) zoomed-in area of the blends prepared by reactive blending at different times, (**c**) derivative of heat flow of the PEF–PET 70–30 samples prepared by reactive blending, and (**d**) the variation of the intrinsic viscosity with reactive blending time.

Transesterification reactions take place when two polyesters are melt-mixed at temperatures above the *T*_m_ of at least one of the polyesters, and this procedure is called reactive blending. Reactive blending usually takes place at temperatures above the *T*_m_ of both blend components. These reactions lead to the formation of block copolymers, or even to the formation of random copolymers if the time of melt mixing is long enough. Reactive blending was applied to the PEF–PET blends at 280 °C, where only PET crystallized. Figure 8a shows the DSC thermograms for the PEF–PET 70–30 blend after different reactive blending times. It is obvious that with increasing blending time the crystallization of the resulting copolymers was suppressed, as revealed by the reduced cold-crystallization enthalpy, while the melting temperature of PET in the blends decreased. Obviously, the crystallization ability was limited due to the transformation of the blend to a copolymer.

Details of the thermograms in the glass transition temperature region are presented in Figure 8b. By increasing the time of reactive blending to 5 min, a single glass transition was eventually observed, indicating the formation of a block copolymer. This is also verified by the single peak observed in the derivative heat flow (Figure 8c). The effect of reactive blending on the molecular weight of the blends was examined by intrinsic viscosity measurements on the blends after different blending times (Figure 8d). As seen in Figure 8d, a small decrease of viscosity occurred during the first 3 min, something that is commonly observed during standard polymer processing procedures. 

**Figure 9:**
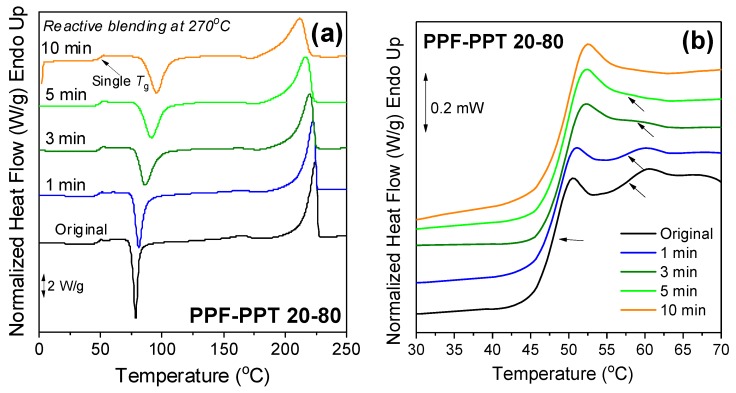

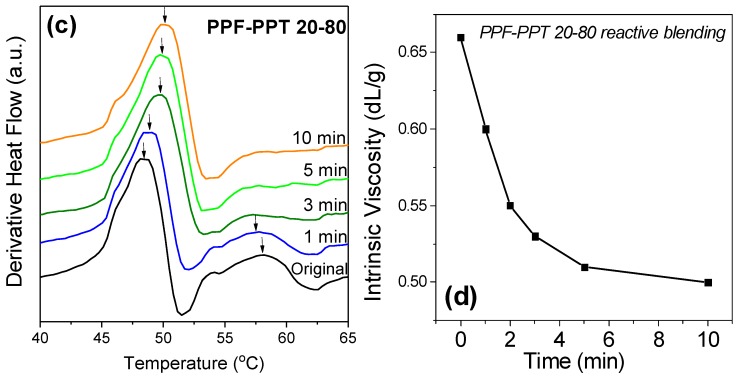
(**a**) Effect of the time of reactive blending on the DSC thermograms of the PPF–PPT 20–80 sample, (**b**) zoomed-in area of the glass temperature for the PPF–PPT sample prepared under different reactive blending times, (**c**) derivative of heat flow, and (**d**) variation of the intrinsic viscosity with reactive blending time.

The effect of reactive blending on the PPF–PPT 20–80 blends was also evaluated in order to assess a potential enhancement of miscibility and dynamic homogeneity. The DSC thermograms of the blends after the application of reactive blending at different times can be seen in Figure 9a. The melting temperature of PPT in the blends decreased with increasing blending time and crystallization was once again suppressed, indicating the formation of a random copolymer. The zoomed-in area of the glass temperature at different reactive blending times can be seen in Figure 9b and the derivative of the heat flow in Figure 9c. Two peaks can be observed up to 3 min of reactive blending, while after 5 and 10 min a single glass temperature appeared. The fact that the *T*_g_ at 5 and 10 min of reactive blending displayed a narrow breadth is an indication of dynamic homogeneity. Finally, the same picture as that seen in the PEF–PET blends was formed in terms of the intrinsic viscosity of the PPF–PPT 20–80 blends, as a small decrease was also recorded with increasing blending times of up to 5 min (Figure 9d). 

### 3.5. Compatibilization of PEF–PET Blends

**Figure 10:**
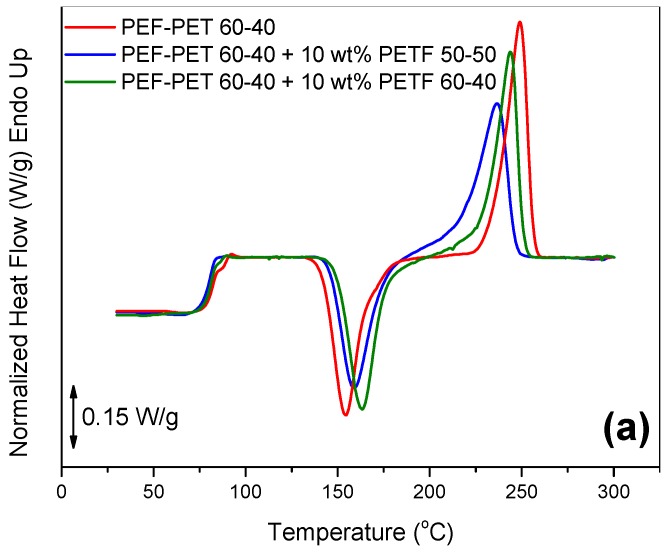

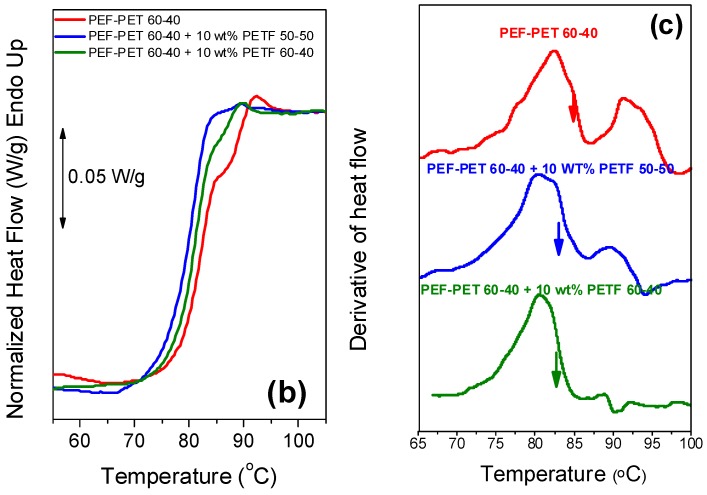
(**a**) DSC thermograms of the PEF–PET 60–40 blends with or without the poly(ethylene terephthalate-*co*-ethylene furanoate) (PETF) copolymer compatibilizer, (**b**) zoomed-in area of the glass temperature, and (**c**) derivative of heat flow for all samples under study.

An attempt was made to further improve the compatibility of the blends by using poly(ethylene terephthalate-*co*-ethylene furanoate) (PETF) random copolymers as compatibilizers. The copolymers were synthesized by the melt polycondensation method, as was described in detail in a previous work [43]. Two copolymers were prepared and used as compatibilizers: PETF 50–50 and PETF 60–40. The PET–PEF 60–40 blend was tested in both cases. The compatibilizer was added at 10 wt % in the blend. DSC thermograms of the PEF–PET 60–40 blend with or without the compatibilizer are shown in Figure 10a. The addition of the compatibilizers resulted in the shift of the high-temperature *T*_g_ towards lower temperatures, suggesting enhanced miscibility (Figure 10b,c). However, miscibility was not complete, as the low-temperature *T*_g_ remained intact. This is suggestive of the presence of: (i) nearly pure PET domains and (ii) mixed domains comprising both PET and PEF segments. The combination of results on the melting point depression and of the shift in the glass temperature of the PEF component in the blends suggest increased miscibility in the presence of compatibilizer, especially when the compatibilizer was the PEFT 50–50 random copolymer.

## 4. Conclusions

In this work, we evaluated whether poly(alkylene furanoate)s can successfully form blends with poly(alkylene terephthalate)s by measuring their glass temperatures and evaluating their structural characteristics. Even though the presence of a single glass transition cannot always be considered as a criterion for miscibility in polymer blends, when the blend components belong to a set of polymers where changes in polarizability from one to another are small, the shifts in the individual *T*_g_s can indicate partial miscibility. On this basis, PEF–PET and PPF–PPT blends displayed a dual glass temperature, indicating the immiscibility of the individual components of the blends. On the other hand, the PCHDMF–PCHDMT blends showed a single glass temperature. However, the differences between the glass temperatures of the components were very small, so this could be the reason for the observation of a single *T*_g_. Overall, the poly(alkylene furanoate)/poly(alkylene terephthalate) blends were immiscible or partially miscible. Next, reactive blending was applied in order to enhance miscibility and it was shown that the PEF–PET and PPF–PPT blends were eventually transformed into copolymers after a specific blending time. Finally, poly(ethylene terephthalate-*co*-ethylene furanoate) (PETF) random copolymer was successfully introduced to the PEF–PET blend in order to enhance the compatibility of the blends.
